# Regional variation in trajectories of healthcare worker infections during the COVID-19 pandemic in Italy

**DOI:** 10.1017/ice.2020.189

**Published:** 2020-05-04

**Authors:** Saverio Bellizzi, Catello Mario Panu Napodano, Paola Salaris, Giuseppe Pichierri, Giovanni Sotgiu

**Affiliations:** 1 Medical Epidemiologist, Independent consultant, Geneva, Switzerland; 2 Infectious Diseases Department, AOU Sassari, University of Sassari, Sassari, Italy; 3 Mater Olbia Hospital, Olbia, Italy; 4 Kingston Hospital NHS Foundation Trust, Microbiology Unit, Kingston Upon Thames, United Kingdom; 5 Department of Medical, Surgical and Experimental Sciences, University of Sassari, Sassari, Italy

*To the Editor*—Healthcare workers (HCWs) have been key in the current global response against the COVID-19 epidemic; their safety can help address the clinical and public health challenges associated with SARS-CoV-2 infection.^1^

As of late March, infections of HCWs in Italy reached a peak of ~10.0% of the total COVID-19 cases,^2^ representing a potential amplifier of the epidemic following the viral transmission within and outside the health facility environment, to HCWs, visitors, inpatients, and outpatients. The Italian National Health Institute (Istituto Superiore di Sanita, ISS), has been issuing a biweekly bulletin on the COVID-19-update since March 19, 2020. These bulletins report on Italian regional data collected by local laboratories, stratified by demographic and epidemiological variables (eg, age, province, etc). Until April 2, bulletins were also reporting the cumulative number of SARS-CoV-2–positive HCWs.^3-7^ Unfortunately, no data on incident infections among HCWs have been published in the most recent reports.

**Fig. 1.**
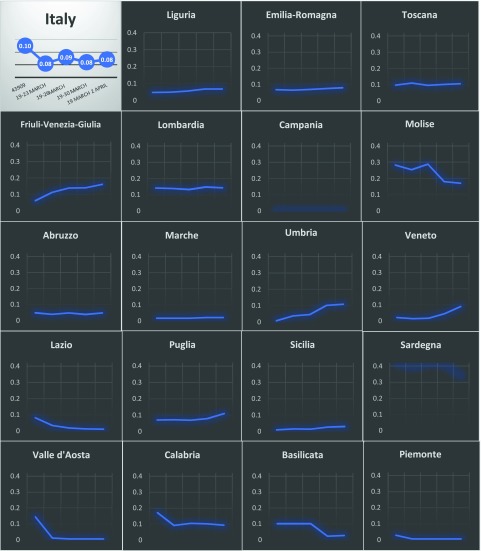
Reporting graphs of proportional nfections in 19 regions in Italy.

The available data show wide regional disparities, both in terms of HCW infection prevalence and in trajectories over the 4 weeks of monitoring (Fig. 1). On March 19, 2020, the cumulative number of positive individuals ranged from 7 in Valle d’Aosta to 19,882 in Lombardy, with a proportional attributable contribution to the total amount of cases varying from 0.2% (n = 1) in Campania to 41.5% (n = 44) in Sardinia. Although the overall national trend of positive healthcare workers out of the total cases showed a slight decrease (from 9.5% to 8.4%) from March 19 to April 2, several regional patterns were also described in the same period (Fig. 1).

Some regions (eg, Piemonte, Valle d’Aosta, Marche, Lazio, Campania, and Basilicata) initially showed low percentages of infected HCWs, and these cases were rapidly and accurately managed. The Molise region recorded a prevalence of 28.1% during the first day of monitoring, which was successfully lowered to 17.0%, whereas Sardinia showed a high percentage (34.0%) at the beginning of April. On the other hand, stable lower estimates were reported in Lombardy (14.0%), Tuscany (10.0%), Calabria (10.0%), Emilia-Romagna (7.0%), Liguria (6.0%), and Abruzzo (5.0%). Sicily and Puglia showed a slight proportional increase (from 0.9% to 3.0% and from 7.0% to 10.9%, respectively) and several areas showed dramatic increases: Veneto (from 2.0% to 8.8%), Friuli-Venezia-Giulia (from 6.2% to 16.1%) and Umbria (from 0.7% to 10.8%).

Cautious interpretation of these data is needed. A rapid decreasing proportion of positive HCWs might be the consequence of a rapid inflation of the total positive cases in the general population or of a strong regional response based on tailored and immediate public health interventions. On the other hand, the proportional stability (~15.0%) might be explained by the high regional burden of infected individuals over the previous weeks (46,071 cumulative cases by April 2). In regions where the proportion of infected HCWs is increasing or is still very high, appropriate public health measures to curb the trend should be implemented and scaled up.

Importantly, the national picture does not clearly showcase regional and provincial scenarios. Regional dynamics are significantly affected by local events, such as incident clusters of disease, as well as adoption and adaptation of national preparedness plans and their appropriate implementation. Furthermore, accurate and continuous monitoring of local data is needed, and the responses to emergencies must be adapted in the most granular way. As emphasized by the recent World Health Organization (WHO) Health Emergency and Disaster Risk Management Framework (HEDRM), monitoring and evaluation is a critical step in the risk management cycle and can be applied in any moment of the continuum, from prevention and mitigation to preparedness, response, and recovery.^8^ Recording and reporting regional data on infections among HCWs should be comprehensively coordinated by the ISS. Reports should be published to provide key feedback to the scientific community to facilitate participation in the fight against SARS-CoV-2.
